# Hexavalent-Chromium-Induced Oxidative Stress and the Protective Role of Antioxidants against Cellular Toxicity

**DOI:** 10.3390/antiox11122375

**Published:** 2022-11-30

**Authors:** Veer Singh, Nidhi Singh, Manisha Verma, Rashmi Kamal, Ritesh Tiwari, Mahesh Sanjay Chivate, Sachchida Nand Rai, Ashish Kumar, Anupama Singh, Mohan P. Singh, Emanuel Vamanu, Vishal Mishra

**Affiliations:** 1 School of Biochemical Engineering, Indian Institute of Technology, Banaras Hindu University, Varanasi 221005, India; 2 Centre of Bioinformatics, University of Allahabad, Prayagraj 211002, India; 3 Department of Biochemistry, Rajendra Memorial Research Institute of Medical Sciences, Patna 800007, India; 4 Centre of Biotechnology, University of Allahabad, Prayagraj 211002, India; 5 Centre for Energy and Environment, Indian Institute of Technology, Patna 801106, India; 6 Faculty of Biotechnology, University of Agricultural Sciences and Veterinary Medicine of Bucharest, 011464 Bucharest, Romania

**Keywords:** hexavalent chromium, source of Cr (VI), Cr (VI) toxicity, reactive oxygen species, antioxidants

## Abstract

Hexavalent chromium is a highly soluble environmental contaminant. It is a widespread anthropogenic chromium species that is 100 times more toxic than trivalent chromium. Leather, chrome plating, coal mining and paint industries are the major sources of hexavalent chromium in water. Hexavalent chromium is widely recognised as a carcinogen and mutagen in humans and other animals. It is also responsible for multiorgan damage, such as kidney damage, liver failure, heart failure, skin disease and lung dysfunction. The fate of the toxicity of hexavalent chromium depends on its oxidation state. The reduction of Cr (VI) to Cr (III) is responsible for the generation of reactive oxygen species (ROS) and chromium intermediate species, such as Cr (V) and Cr (IV). Reactive oxygen species (ROS) are responsible for oxidative tissue damage and the disruption of cell organelles, such as mitochondria, DNA, RNA and protein molecules. Cr (VI)-induced oxidative stress can be neutralised by the antioxidant system in human and animal cells. In this review, the authors summarise the Cr (VI) source, toxicity and antioxidant defence mechanism against Cr (VI)-induced reactive oxygen species (ROS).

## 1. Introduction

Environmental contamination is defined as the elevated concentration of unwanted materials in air, water and soil beyond the permissible limit. It is also defined as an undesirable change in the natural environment that has harmful effects on both animals and plants [1]. Heavy metals are naturally occurring elements having a higher density as compared to water [2]. Chromium (Cr), arsenic (As), lead (Pb), iron (Fe), cadmium (Cd), nickel (Ni), mercury (Hg) and cobalt (Co) metallic ions are toxic even in low quantities [3]. These heavy metals become lethal when their intake is in excess and when they are not metabolised by the body and accumulate in the intra- or extracellular space of body organs [4,5]. Heavy metals have been found in the body parts of fishes captured from the metal-contaminated aquatic system [6]. Heavy metals enter the human body via the food chain [7]. The effluent emanating from tanneries; battery-manufacturing units; glass, paint and metal plating industries; and pigment and steel productions cause heavy metal pollution in terrestrial and aquatic ecosystems [8]. Coal has an important application in the energy sector and is considered a major energy source in India, China, Nepal, Pakistan and other countries. It is an easily available and inexpensive source of energy compared to other energy sources. These features make it a priority choice for several industries. During coal processing, a large volume of toxic substances is released as coal washery effluent (CWE), including heavy metal ions, such as Cd (II), Cr (VI) and Pb (II), which massively contaminate the natural environment [9].

Cr (VI) has the seventh rank in the ATSDR [10]. It is considered a priority carcinogen, which is generated from several anthropogenic activities, such as tanning of leathers, pigment and rubber production, paint manufacturing, production of anticorrosion agents and processing of coal [11]. Cr (VI) is 100 times more toxic as compared to Cr (III). High solubility in water and a higher oxidative state make Cr (VI) more lethal. The Central Pollution Control Board (CPCB), India, has defined the maximum allowable concentration of Cr (VI) in the effluent of industrial units as 1.0–2.0 mg/L [12]. Cr (VI) enters the human body and causes cancer, liver and kidney damage and difficulties in respiration [13]. The World Health Organization (WHO) has recommended a permissible limit of up to 0.05 mg/L of Cr (VI) in drinking water [14].

Several cell surface receptors are actively involved in the intake of Cr (VI) ions inside the cell [15]. Cr (VI) ions in the intracellular space of the cell either bind with metallothionein proteins or are transformed into a less toxic form. Animal cells use intracellular Cr (VI) ions as electron acceptors or detoxify them by producing soluble enzymes [15,16]. Reactive oxygen species (ROS) are produced as an intermediate component during the reduction of Cr (VI) to Cr (III) [17]. ROS are highly reactive, which damages cell organelles, and these Cr (VI)-mediated ROS can be neutralised by the antioxidant system [18]. Antioxidants of the cell minimise Cr (VI) toxicity and protect the cell organelles from Cr (VI)-mediated oxidative damage [15,19]. Thus, antioxidant activity of the animal cell actively participates in the detoxification of Cr (VI) toxicity [20].

This review is focused on Cr (VI) contamination in wastewater, its source and Cr (VI)-induced toxicity in humans and animals. The generation of Cr (VI)-induced ROS in the animal cell and their toxicity are discussed in this review. Additionally, the expression of antioxidants and their role in the detoxification of Cr (VI)-induced toxicity are summarised in this review.

## 2. Source of Cr (VI) Contamination

**Figure 1:**
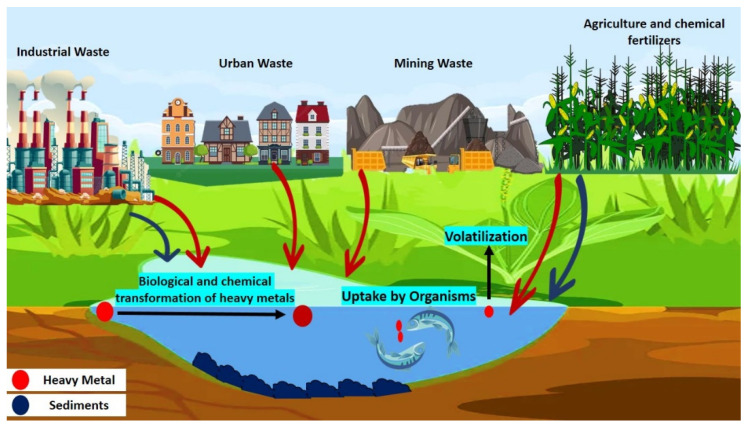
Anthropogenic sources of Cr (VI).

Chromium is a common element present in the earth’s crust in its natural form. Chromium is generally extracted from nature in the form of chromate ore (FeCr_2_O_4_). The anthropogenic source of Cr (VI) in the environment is mostly untreated or partially treated industrial effluent discharge in surface water sources [21]. There are several industrial units, such as electroplating, leather tanning, steel processing, wood preservation, paint industry and mine tailings, which are major sources. The sources of Cr (VI) contamination in the environment are shown in Figure 1.

Chromium exists in several oxidation states, such as Cr^1+^, Cr^2+^, Cr^3+^, Cr^4+^, Cr^5+^ and Cr^6+^. Cr^3+^ and Cr^6+^ are the most stable forms of chromium [22]. Cr^3+^ or Cr (III) mainly exists in the form of Cr(OH)^2+^, Cr(OH)_3_, Cr(OH)_4_^−^ and Cr(OH)_5_^2−^. Cr (VI) exists in the form of three major ionic species: CrO_4_^2−^, HCrO_4_^−^ and Cr_2_O_7_^2−^ [23].

Recent studies have shown that Cr (VI) is highly soluble in water and is more than 100 times lethal than Cr (III) [21]. The toxicity of Cr (VI) is due to its higher oxidation state and solubility in water. The exposure of Cr (VI) in humans and animals is mainly through drinking of Cr (VI)-contaminated water, ingestion of contaminated food and direct skin contact [22]. Continuous accumulation of toxic Cr (VI) along food chains leads to its biomagnification, which could put human lives in danger by causing lung cancer, dermatitis, kidney and gastrointestinal impairment and exasperation to the respiratory tract and eyes [24]. Cr (III) is considered a non-toxic and essential micro-nutrient in mammalian diets that helps in the metabolism of glucose and lipids [25].

## 3. Cr (VI)-Induced Oxidative Stress and Disruption of Cell Components

**Figure 2:**
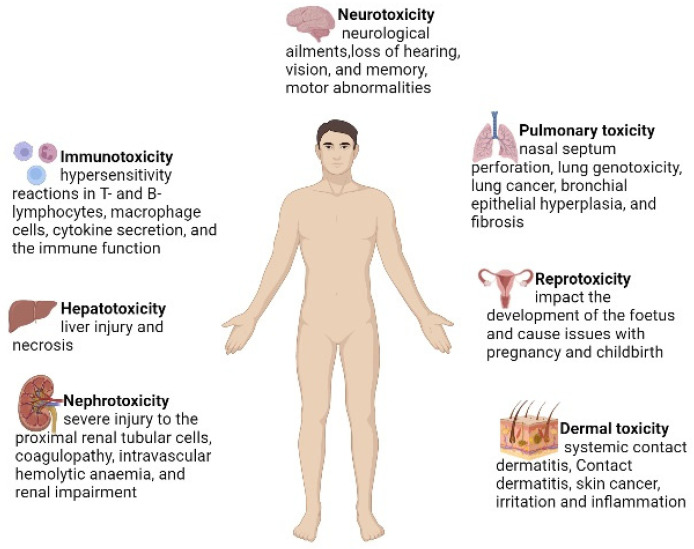
The major hazardous effects of Cr (VI) on human health.

Cr (VI) is a well-known toxic agent, and its toxicity highly depends on the oxidation states and ionic species [26]. Cr (VI) has higher solubility and reactivity as compared to Cr (III). It can easily cross the plasma membrane and enter the cell compartment through several cell surface receptor phosphate transporters and anion transporters [27]. Cr (VI) is more hazardous due to its higher oxidative state and solubility [28]. Cr (VI) has a negative impact on human health, which responsible for the damage of several organs, including the lungs, liver and kidneys [29]. The lethal effects of Cr (VI) are listed in Figure 2.

Several studies have reported that Cr (VI) causes carcinogenicity and multiple-organ damage, such as liver and cardiac failure and renal damage [30]. Gumbleton and Nicholls (1988) reported that Cr (VI) induces kidney damage in rats after a sub-cutaneous injection of Cr (VI). Bagchi et al. [31] reported that Cr (VI) induces hepatic mitochondrial, microsomal lipid peroxidation and increases lipid metabolites in the urine when Cr (VI) is administrated orally. Other important toxic effects of Cr (VI) are respiratory cancer, chromosomal abnormalities and DNA strand breaks [32].

The reduction of Cr (VI) is considered a detoxification process when it occurs at a distance from the nucleus and other cell organelles or outside the cell. If Cr (VI) reduction occurs within the cell, it induces oxidative-mediated toxicity and damages cell organelles, and mutation in the DNA take place [33]. In case Cr (VI) is converted into Cr (III) outside the cell, the reduced Cr (III) and other intermediate components are unable to transport into the cell compartment and hence a toxic effect is not observed [34]. Cr (VI) passes through the cell membrane and enters the intracellular space and is subsequently reduced to Cr (III) [35]. During the reduction process, ROS are generated, which cause cell toxicity. Studies suggest that Cr (VI) toxicity is mainly due to an increase in ROS production, which are produced by the Fenton reaction [36,37]. The generation of ROS in different cell lineages and their lethal impacts are mentioned in Table 1.

The toxicity of Cr (VI) is directly proportionate to its dosage and exposure time. Cr (VI) induces transcriptional upregulation of apoptosis-related genes, such as p53 and caspase (3 and 9), and downregulates genes involved in the antioxidant pathway [38]. Long-term exposure to Cr (VI) causes higher oxidative stress in the cell and induces ROS-mediated cell death [48].

Intracellular ROS cause DNA, RNA, protein and mitochondrial damage. The main mechanism by which Cr (VI) shows toxicity is through the disruption of transcriptional regulation, which makes it challenging to have normal gene expression pathways as complexation and DNA changes are more common in regions of active DNA replication and transcription [49]. Several in vitro and in vivo studies have revealed that Cr (VI) induces oxidative stress by enhancing the production of ROS, which leads to deterioration of lipids and enzymes and DNA damage [45]. In the cell, a cascade of cellular events occurs following Cr (VI)-induced oxidative stress, including enhanced generation of superoxide anions and hydroxyl radicals. In addition, Cr (VI)-induced oxidative stress is also responsible for increased lipid peroxidation, activation of protein kinase C, DNA fragmentation, alteration in gene expression, modulation of intracellular oxidised states and apoptotic cell death [50]. Holmes et al. [51] reported that Cr (VI) can inhibit the DNA replication and repair process. Bagchi et al. [45] investigated the dose-dependent effects of Cr (VI) in female C57BL/6Ntac and p53-deficient C57BL/6TSG p53 mice. The authors observed the enhanced production of ROS and lipid peroxidation in the hepatic and brain tissues of female mice. Son et al. [52] reported Cr (VI)-induced cell death of mouse skin epidermal cells by apoptosis or necrosis. Cr (VI)-induced cell death is a Cr (VI) dose-dependent phenomenon. Authors reported that several activities occur when cells are exposed to Cr (VI), such as cell shrinkage, migration of cells into the sub-G1 phase and increase in annexin V positively. The presence of Cr (VI) in culture media increases mitochondrial membrane depolarisation and caspase activation. Son et al. [52] suggested that Cr (VI) induces mitochondrial-mediated and caspase-dependent apoptosis in mouse skin epidermal cells through activation of p53.

**Figure 3:**
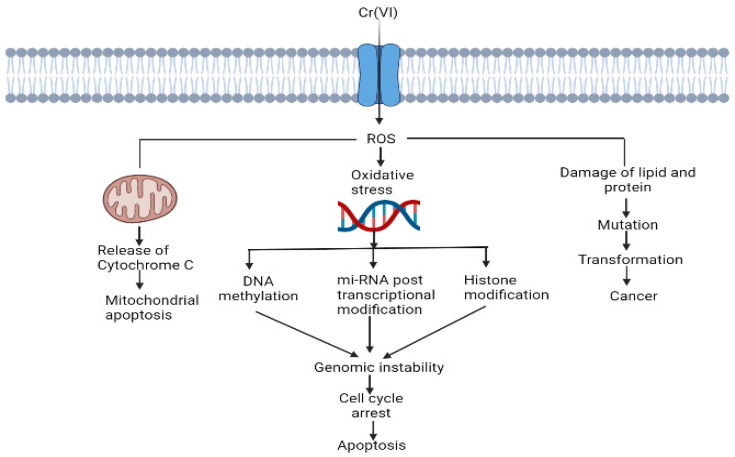
Cr (VI)-induced ROS generation and their role in the disruption of cell organelles.

ROS-mediated disruption of cell organelles is represented in Figure 3.

Cr (VI) alters histone modifications, miRNA expression and DNA methylation [44]. Cr (VI) causes mitochondrial damage by oxidising Trx. For proper functioning of the cell, Trx should be in the reduced state, and the Trx system maintains a regular thiol redox balance and plays a role in cell survival [53].

## 4. Antioxidants and Their Protective Role against Cr (VI) Toxicity

ROS include nitric oxide, superoxide anions, hydroxy radicals and hydrogen peroxide. ROS have high chemical reactivity, which causes protein damage, DNA mutation and lipid peroxidation. In usual circumstances, the antioxidant system reduces the uneasiness triggered by ROS in the cell [54]. Increased ROS take over the antioxidants present in the cell and generate oxidative stress. Biomolecules involved in cell signalling and regulation are extremely sensitive to the redox state of the cell [55]. Antioxidants prevent the oxidation of biomolecules inside cells by activating defensive enzyme/proteins and scavenging superoxide. Lower antioxidant levels in aerobic organisms might lead to carcinogenicity, mutagenicity or cytotoxicity. The antioxidant systems (enzymatic, polyphenolic, endogenous, exogenous) are considered a new tool for ROS detoxification [56].

### 4.1. Enzymatic Antioxidants

#### 4.1.1. Superoxide Dismutase (EC 1.15.1.1)

Superoxide dismutase (SOD) stubs out superoxide anions by altering them to peroxide, which is further wiped out by catalase or glutathione peroxidase [57]. In aerobic respiration, a low concentration of superoxide is generated constantly [54]. In mitochondria, the electron transport chain transfers four electrons to oxygen to form water. Sometimes, the electron transport chain leaks a single electron, which forms superoxide instead of water. Superoxide releases iron and reduces Fe (III) to Fe (II). Fe (II) reacts with H_2_O_2_ and generates hydroxyl radicals. SOD alters superoxide into H_2_O_2_ and O_2_ [54].

In human beings, in total, three forms of SOD have been recognised: (i) cytosolic Cu, Zn-SOD; (ii) extracellular-SOD; and (iii) mitochondrial SOD. Four classes of SOD have been recognised on the basis of cofactors, having either mononuclear Fe/Mn/Ni or binuclear Cu/Zn [57,58,59].

SOD regulates inflammation, lipid metabolism, oxidation in cells and oxidative stress. SOD prevents lipid droplet formation, oxidation of lipoprotein in macrophages and lipid peroxidation [54]. SOD is an effective antioxidant that reduces Cr (VI) chronicity and protects against oxidative-damage-related lethal effects. Table 2 presents disease management using SOD enzymes.

#### 4.1.2. Catalase (EC 1.11.1.6)

The catalase enzyme has a molecular mass of 240 kDa and consists of 4 identical subunits (ferriprotoporphyrin groups, 60 kDa) arranged tetrahedrally (tetrameric haem enzyme). Catalase is a highly efficient enzyme as it remains unsaturated at any concentration of hydrogen peroxide [70]. Catalase counters H_2_O_2_ and generates molecular oxygen, water and H donors (formic acid, ethanol, methanol, phenol). In animals, hydrogen peroxide is broken down enzymatically by catalase and glutathione peroxidase. Still, catalase is not crucial for several cells in regular conditions, and it helps to attain tolerance towards oxidative (stress) conditions in the adaptive response of the cell [71]. Catalase inhibits drug-induced consumption of oxygen, and hence, catalase-enriched cells have amplified sensitivity towards paraquat, bleomycin and adriamycin. Catalase catches hydrogen peroxide and catabolises it to oxygen to maintain the oxygen concentration for repeated cycles of chemical reduction [56].

#### 4.1.3. Glutathione Peroxidase (EC 1.11.1.19)

Glutathione peroxidase (a selenium-containing peroxidase) catalyses the reduction of hydroperoxides (H_2_O_2_ and ROOH) using glutathione and prevents oxidative damage in mammalian cells [57]. Usually, glutathione peroxidase works with catalase to break down H_2_O_2_; however, glutathione peroxidase is itself sufficient to counter lipids and other organic hydroperoxides effectively. Glutathione peroxidase works against lower oxidant stress levels, while catalase is significant against severe levels of oxidant stress [56]. In human erythrocytes, glutathione peroxidase is the principal antioxidant enzyme as catalase has lower affinity towards hydrogen peroxide as compared to glutathione peroxidase [56]. In total, five glutathione peroxidase isoenzymes have been found in mammals. Even though their expression is similar, yet the expression levels of each isoform differ based on the type of tissue. Cytosolic glutathione peroxidase and mitochondrial glutathione peroxidase (GPX1) reduce H_2_O_2_ and fatty acid hydroperoxides by using glutathione. GPX1 has four identical subunits, and each subunit contains one selenocysteine residue [72]. Cytosolic glutathione peroxidase (GPX1) and phospholipid hydroperoxide glutathione peroxidases (PHGP) are found in most tissues [73]. PHGP is found in the membrane and cytosol both. PHGP can reduce cholesterol hydroperoxides, phospholipid hydroperoxides and fatty acid hydroperoxides generated in oxidized lipoproteins and peroxidised membranes [72,73]. Cytosolic glutathione peroxidase is mostly found in the liver, kidneys and erythrocytes. PHGP is well expressed in the testes and renal epithelial cells. A selenium-independent glutathione peroxidase (GPX5) has been found in the mouse epididymis [74]. Extracellular glutathione peroxidase and cytosolic glutathione peroxidase are rarely present in many tissues but are found in the gastrointestinal tract and kidneys, respectively [72,73].

### 4.2. Endogenous and Exogenous Antioxidants

Endogenous antioxidants are the by-products of human metabolism. An endogenous antioxidant may or may not be enzymatic [74]. Superoxide dismutase is one of the enzymatic antioxidants involved in the first line of defence. Peroxiredoxins, catalase, glutathione reductase and glutathione peroxidase are some other important enzymatic endogenous antioxidants in the first line of defence [74]. These enzymes neutralise hydrogen peroxide, yielding oxygen and water. Ceruloplasmin, ferritin, transferrin and albumin are examples of nonenzymatic molecules that participate in the body’s first line of defence and are preventive antioxidants found in the plasma [74]. These proteins bind to transition metal ions to prevent the development of new reactive species. Additionally, metallothionein is also crucial for protecting against reactive species. It has a large number of –SH groups as the source of its main antioxidant effects [74]. Many endogenous antioxidant mechanisms in living beings depend on exogenous antioxidants, such as polyphenols, carotenoids, vitamin E and vitamin C. To maintain redox homeostasis, endogenous and exogenous antioxidants work together synergistically [75]. Examples include prevention of the lipid peroxidation process, which can cause damage to the cell membrane [75]. Regeneration of vitamin E by vitamin C or glutathione can prevent the lipid peroxidation process, thus protecting cell from membrane damage [75].

Many researchers agree that the synthesis of Cr (III) from the intracellular reduction of Cr (VI) and the subsequent oxidation of intracellular macromolecules (e.g., DNA) are essential. Indeed, it could be the cause of chromium’s carcinogenicity and mutagenicity [76]. Intracellular reduction of Cr (VI) can be either enzymatic or non-enzymatic, depending on the tissues and other physiological parameters [76]. The enzymatic reduction of Cr (VI) requires enzymatic antioxidant systems, such as nicotinamide adenine dinucleotide phosphate reductase, cytochrome c reductase, P450 cytochrome, catalase, glutathione S-transferase, glutathione peroxidase, glutathione reductase, superoxide dismutase, thioredoxin reductase, heme oxygenase biliverdin reductase and DT-diaphorase [76].

### 4.3. Polyphenolic Antioxidants

3,4-Dihydroxybenzaldehyde (DHB), commonly known as protocatechualdehyde, is an antioxidant that protects the cell from Cr (VI) toxicity. Erythrocytes and lymphocytes were used for this investigation because DHB is both cyto- and geno-protective [77]. The defence provided by DHB against inactivation by Cr (VI) may be a result of its inherent antioxidant characteristic, quenching the free radicals and ROS/RNS created by this metal ion, or it may be a result of DHB’s incorporation into the erythrocyte membrane, where it can affect the properties of enzymes/proteins [78]. DHB prevents oxidative-stress-related cell death by preventing oxidative DNA damage and apoptosis [35].

Ellagic acid is a well-known phenolic antioxidant and plays an important role against oxidative stress. Several studies were conducted on six groups of male Wistar rats to determine how renal damage caused by Cr (VI) works. It was found that ellagic acid shields rats treated with Cr (VI) that caused ROS stress in their renal tissues [79]. Ellagic acid enhances glomerular filtration, tubular reabsorption and secretion processes. It also reverses histological alterations brought on by Cr (VI), lowers oxidative stress markers and boosts the activity of antioxidant enzymes [80]. Ellagic acid may be able to chelate Cr^2+^ and Cr^3+^ intermediates, stopping cyclic Fenton/Haber–Weiss reactions since it chelates divalent cations [81].

## 5. Mechanism and Mode of Action of Antioxidants

Cr (VI) is the most toxic heavy metal ion present as an anthropogenic source in water and soil [82]. Cr (VI) induces the production of ROS in the cell, and this oxidative stress is responsible for cell toxicity. The concentration of oxidants in response to the damage caused to fundamental building blocks, such as nucleic acids, lipids, proteins and sugars of the cell increases, ultimately causing detrimental modifications to cellular signalling pathways; for example, the latter leads to mitochondrial dysfunction because of the activation of Ras, Myc, p53 protein and downstream signalling of NF-κB, STAT3, etc., that promotes gene expression during an inflammatory response [83].

To overcome all such detrimental impacts on cellular health, there exists a wide variety of essential antioxidants classified based on their molecular size, functional group activity, origin (endogenous or exogenous), source (natural or synthetic), solubility (hydrophobic or hydrophilic), polarity and physical location (intracellular or extracellular), helping us to understand their interaction behaviour in a particular situation [84]. Antioxidants are an important class of molecules that help prevent oxidative damage to cell components caused by life-threatening heavy metals and their metallic nanoparticles. While each type of antioxidant has its own pros and cons, all follow either action to slow down/neutralize the reactivity of various oxidants so that no further downstream reaction occurs.

1. Chain-break mechanism, in which antioxidants obstruct the oxidation processes by scavenging free radicals. Flavonoids; vitamins, such as vitamins C, E and A; and non-protein endogenous antioxidants, such as albumin, bilirubin and ubiquinol, follow this mechanism [85].

2. Preventive mechanism, in which antioxidants chelate the transition metal or quench the free radical species and decompose them into a non-radical product, hence preventing the chain initiation step. Ceruloplasmin and albumin as Cu ion sequesters, transferrin and lactoferrin as Fe sequesters, citric acid, EDTA, carotenoids and glutathione peroxidase follows this mechanism [86].

3. Synergetic mechanism, in which one antioxidant couple works with another in synergy. Together, they are more effective than a single antioxidant alone. For example, a combination of tocopherol with citric acid has a synergetic effect [85].

The cellular protective mechanisms against Cr (VI)-induced ROS consist of multiple antioxidants, such as enzymatic, polyphenolic, endogenous and exogenous antioxidants [87,88].

The antioxidants can be divided into two types:

(1) React with ROS and decrease their level in the cell. SOD, catalase and peroxidase are major antioxidants considered in the category.
(2) Glutathione reductase (GR) is one of the well-known antioxidants of the group. This type of antioxidants restores the reduced forms of non-enzymatic antioxidants (GSH) [87].

Kamran et al. [89] investigated Cr (VI) toxicity and the antioxidant-mediated protective mechanism in the choysum (*Brassica parachinensis* L.). The authors observed that antioxidant enzyme, ascorbate and glutathione pool production was induced when the plant is exposed to Cr (VI). The antioxidant system in the plant suppressed the Cr (VI)-mediated oxidative stress in the plant.

In recent years, apart from endogenous antioxidants, the research focus is also shifting towards exploring the antioxidant potential of exogenous non-proteinaceous, non-enzymatic lipophilic antioxidants because these can penetrate blood lipoproteins and cell membranes to maintain prolonged high bioavailability, protecting the cell membrane from Cr (VI)-induced oxidative stress [86].

Phenolic compounds in free, conjugated or bound form; polyphenols; condensed tannins; fatty acids; carotenoids; flavonoids; tocopherols; and tocotrienols are known classes of lipophilic antioxidants [86]. Polyphenols, such as resveratrol and luteolin, significantly inhibit the activation of caspase-3 and modulate mitogen-activated protein kinases, which play an important role in neuronal apoptosis [83].

## 6. Antioxidant Measurement Methods

Based on chemical reactions, antioxidant capacity can be divided into two categories.

### 6.1. HAT-Based Assays

In this, the free-radical-scavenging capacity is measured instead of the antioxidant capacity, in which the hydrogen atom (H^−^) of phenolic antioxidants is transferred to an ROO^−^ radical and stabilised by resonance. Since the fluorescent probe and the antioxidant both react with ROO^−^, competitive decay of the probe is observed in the absence and presence of the antioxidant.

As an example, oxygen radical absorbance capacity (ORAC) assay, hydroxyl radical averting capacity (HORAC) assay and total peroxyl-radical-trapping antioxidant parameter (TRAP) assay are HAT-based assays [82].

### 6.2. ET-Based Assays

These are relatively slower, pH- and solvent-dependent assays. The antioxidant action is initiated with a suitable redox-potential probe, i.e., the antioxidant reacts with a fluorescent or coloured probe (oxidising agent) instead of peroxyl radicals. The degree of colour change (either an increase or a decrease) of absorbance at a particular wavelength is correlated with the concentration of antioxidants in the sample. Ferric-reducing antioxidant power (FRAP) assay, thiobarbituric-acid-reactive substances (TBARS) assay and Trolox-equivalent antioxidant capacity (TEAC) assay are a few ET-based assays [82]. A number of protocols based on these two mechanisms are tabulated in Table 3.

## 7. Conclusions

Water contamination is a major issue worldwide. Heavy metal ions, such as Cr (VI), are release from several industrial processes and discharge into water bodies. These Cr (VI) ions enter the food chain and cause several health issues in humans and other animals. The major health issues due to Cr (VI) contamination are kidney damage, liver failure, cardiac disorders, pulmonary disorders and reproduction complications. The higher toxicity of Cr (VI) is due to its oxidation state and solubility. Cr (VI) can easily cross the cell membrane and enter the cell. Intracellular Cr (VI) is gradually reduced to Cr (III) and generates ROS during reduction as a reaction intermediate. ROS can damage cell components, such as DNA, RNA, protein and mitochondria. These ROS can be neutralised by several antioxidants, such as SOD, catalase and glutathione peroxidase, present in the cell. The antioxidants in animals create a defensive mechanism, which can protect the cell from oxidative stress. In this review, we discussed that Cr (VI) contamination, Cr (VI) toxicity and the antioxidant-mediated defence mechanism against Cr (VI)-mediated oxidative stress.

## Figures and Tables

**Table 1:** Cr (VI)-induced ROS generation and their lethal impacts.

Cell Type	Impact	Citation
Cardiomyocytes of broiler	Apoptosis-related genes Bax and p53 expression, mitochondrial malfunction and oxidative stress, myocardial apoptosis and autophagy	[38]
Liver cell of zebrafish	Downregulation of the Bcl2 gene and transcriptional activation of apoptosis-related p53, Bax, caspase 9 and caspase 3 genes, which changes the elemental composition of the liver	[39]
Liver and kidney cells of Sprague-Dawley rats	Dose- and time-dependent effects induced DNA damage due to increase in ROS levels	[40]
Hepatocytes (HepG2) of humans	Mitochondrial damage, apoptosis, oxidative stress	[41]
Lung epithelial cells of rats	ROS-induced cell death and activation of the p53-related pathway	[42]
Liver and kidney cells of carassius auratus	Oxidative stress, genotoxicity and histopathology	[43]
Bronchial epithelial cells (Beas-2B) of humans	Cell transformation	[44]
Liver and brain tissues of mice	Oxidative stress and tissue damage	[45]
Gill and kidney cells of Anguilla anguilla L.	Genotoxicity at a higher concentration in gills and at all concentrations in kidneys	[46]
Kidney cells of Wistar rats	Apoptosis and autophagy, oxidative stress in kidneys, mitochondrial dynamics disorder via inhibiting the sirt/pgc-1a pathway	[47]

**Table 2:** Human disease management using SOD enzymes.

Disease	Role of Superoxide Dismutase	References
Cancer	SOD mimetics, such as MnTnBuOE-2-PyP^5+^, play a role in stimulation or work as tumour necrosis factor, which is associated with apoptosis.	[60]
Skin	SOD mimetics could encourage the recovery of wounds, reduce scars and reduce pigmentation of the skin caused by ultraviolet rays.	[55]
Aging attenuation	SOD restores cognitive impairments caused by aging.	[61]
Cystic fibrosis	SOD reduces the pro-inflammatory stimulus.	[62]
Diabetes	SOD treatment reduces oxidative stress in the liver. An SOD mimetic Mn-II (pyane) C12 has been found effective in diabetes. Chemically modified SODs, such as carboxymethyl cellulose-SOD, have been found effective in treating diabetes.	[63,64,65,66]
Neurodegenerative diseases	Cu/Zn-SOD manages oxidative stress in Alzheimer’s and Parkinson’s diseases.	[67,68]
Rheumatoid arthritis	SOD activity reduces rheumatoid arthritis by lipid peroxidation in mitochondria, and SOD processing via liposomes has been found effective against arthritis development.	[54,69]

**Table 3:** Measurement of antioxidants.

Assay	Mechanism	Absorbance (nm)	References
Oxygen radical absorbance capacity (ORAC)	HAT	443 nm, pH = 7.0–7.5	[90,91]
Ferric-reducing antioxidant potential (FRAP)	ET	593 nm, pH = 3.6	[92]
Cupric-reducing antioxidant capacity (CUPRAC)	ET	450/490 nm, pH = 7	[93]
Potassium-ferricyanide-reducing power assay (PFRAP)	ET	700 nm, pH = 6.6	[94]
Ferrous oxidation-xylenol orange (FOX) assay	ET	550 nm	[95]
N,N-dimethyl-p-phenyl-diamine (DMPD)	HAT	517 nm, pH = 5.25	[96]
Thiobarbituric-acid-reactive substances (TBARS) assay	ET	532 nm, pH = 4	[97,98]
Total peroxyl-radical-trapping antioxidant parameter (TRAP)	HAT	Abs. = lambda excitation = 485, lambda emission = 538, pH = 7.4	[90]
